# Restrictive Spirometric Pattern and Postoperative Pulmonary Complications Following Non-cardiothoracic Surgery

**DOI:** 10.1038/s41598-019-49158-1

**Published:** 2019-09-04

**Authors:** Sun Hye Shin, Beomsu Shin, Danbee Kang, Juhee Cho, Hyung Koo Kang, Hae Ri Chon, Jung Soo Kim, Hye Yun Park, Hyun Lee

**Affiliations:** 1 Division of Pulmonary and Critical Care Medicine, Department of Medicine, Samsung Medical Center, Sungkyunkwan University School of Medicine, Seoul, South Korea; 2 Department of Internal Medicine, Yonsei University Wonju College of Medicine, Wonju, South Korea; 3 Center for Clinical Epidemiology, Samsung Medical Center, Sungkyunkwan University School of Medicine, Seoul, South Korea; 4 Department of Epidemiology and Medicine, and Welch Center for Prevention, Epidemiology, and Clinical Research, Johns Hopkins Bloomberg School of Public Health, Baltimore, Maryland USA; 5 Department of Clinical Research Design and Evaluation, SAIHST, Sungkyunkwan University, Seoul, South Korea; 6 Division of Pulmonary and Critical Care Medicine, Department of Internal Medicine, Ilsan Paik Hospital, Inje University College of Medicine, Goyang, Gyeonggi South Korea; 7 Department of Pulmonary Medicine, Osan Hankook Hospital, Osan, Gyeonggi South Korea; 8 Division of Pulmonary and Critical Care Medicine, Department of Internal Medicine, Inha University College of Medicine, Incheon, South Korea; 9 Division of Pulmonary Medicine and Allergy, Department of Internal Medicine, Hanyang University College of Medicine, Seoul, South Korea

**Keywords:** Outcomes research, Risk factors

## Abstract

Despite a substantial population of patients with a restrictive spirometric pattern, few studies have evaluated postoperative pulmonary complications (PPCs) after non-cardiothoracic surgery in these patients. We conducted a retrospective cohort study of 681 adults with a normal or restrictive spirometric pattern who were referred for preoperative evaluation of PPC risk before non-cardiothoracic surgery between March 2014 and January 2015. Overall, 8.7% (59/681) of study participants developed a PPC following non-cardiothoracic surgery. The occurrence of PPCs in patients with a restrictive spirometric pattern was higher than that in those with normal spirometry (12.4% [35/282] vs. 6.0% [24/399], *P* = 0.003). The occurrence of PPCs increased across the categories of restrictive spirometric pattern severity (6.0% with a normal spirometric pattern vs. 6.5% with a mild restrictive spirometric pattern [60 ≤ forced vital capacity (FVC) < 80% predicted] vs. 21.2% with a moderate-to-severe restrictive spirometric pattern [FVC < 60% predicted], P for trend test < 0.001). The length of hospital stay (*P* for trend = 0.002) was longer, and all-cause mortality at 30 days (*P* for trend = 0.008) and 90 days (*P* for trend = 0.001) was higher across the restrictive spirometric pattern severity. In multivariable-adjusted analyses, a moderate-to-severe restrictive spirometric pattern was associated with a higher risk of PPCs compared with a normal spirometric pattern (adjusted odds ratio 2.64, 95% confidence interval 1.22–5.67). The incidence of PPCs in patients with a restrictive spirometric pattern was higher than that in those with a normal spirometric pattern, especially in patients with a moderate-to-severe restrictive spirometric pattern. Patients with a moderate-to-severe restrictive spirometric pattern should be regarded as high risk for developing PPCs following non-cardiothoracic surgery.

## Introduction

Pulmonary complications are a major cause of morbidity and mortality following adverse perioperative events in patients undergoing non-cardiothoracic surgery^1–5^. With recent advances in anesthetic and surgical techniques and improved perioperative care, patients who were previously ineligible are now able to undergo many types of operations. To meet their medical needs, respiratory physicians perform in-depth consultations to evaluate the risk of postoperative pulmonary complications (PPCs) and to determine optimal perioperative management.

A restrictive spirometric pattern is characterized by matched deficits in forced expiratory volume in 1 s (FEV_1_) and forced vital capacity (FVC) with a preserved FEV_1_/FVC ratio^6^. Classically, a restrictive spirometric pattern was associated with interstitial lung disease, neurologic disorders, and space-occupying lesions^7^. However, it is now known that a restrictive spirometric pattern is also associated with a multitude of clinical conditions beyond the lungs and thorax; these include aging^8,9^, smoking^10^, obesity, metabolic syndrome, diabetes mellitus^11–14^, and cardiovascular diseases^15–18^, some of which may increase the risk of PPCs following non-cardiothoracic surgery^1–5^. In addition, comorbid conditions or the illness itself could be the cause of restrictive lung function.

Given that chronic obstructive pulmonary disease (COPD) is the leading cause of PPCs, most previous studies have focused on the relationship between airflow limitation and the occurrence of PPCs following non-cardiothoracic surgery^1,3–5,19,20^. The incidence of PPCs following non-cardiothoracic surgery in patients with COPD ranges from 15% to 25%^5,19,20^, and the severity of airflow limitation in these patients is associated with a greater risk of PPCs after non-cardiothoracic surgery^19,20^. Regarding the relationship between a restrictive spirometric pattern and PPCs, a low FVC has been associated with PPCs following non-cardiothoracic surgery^2,21–24^. However, these studies neither clearly assessed spirometric impairment as “restrictive” nor provided information about the incidence of PPCs based on their severity^2,21–23^. In addition, previous studies that included only those patients undergoing abdominal surgery reported conflicting relationships between restrictive spirometric patterns and PPCs^19,24^.

Therefore, the objective of the present study was to evaluate the incidence of PPCs in adult patients with a restrictive spirometric pattern who underwent non-cardiothoracic surgery and the association between the severity of the restrictive spirometric pattern and the occurrence of PPCs following non-cardiothoracic surgery.

## Methods

### Participants

**Figure 1:**
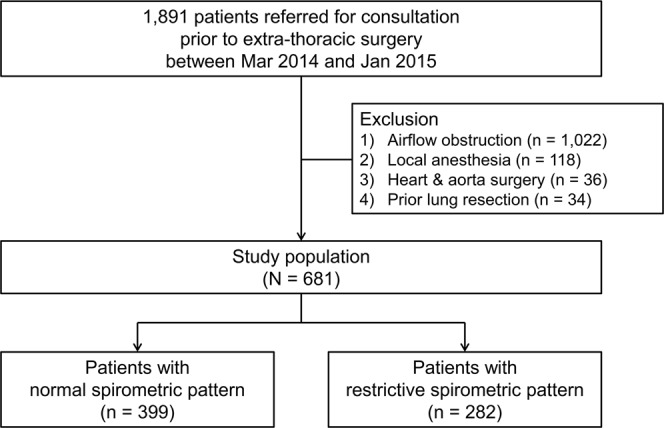
Flow diagram. Airflow obstruction was defined as pre-bronchodilator FEV_1_/FVC < 0.7. Normal spirometry was defined as FEV_1_/FVC ≥ 0.7 and FVC ≥ 80% predicted. Restrictive spirometric pattern was defined as FEV_1_/FVC ≥ 0.7 and FVC < 80% predicted. *FEV*_1_ forced expiratory volume in 1 second, *FVC* forced vital capacity.

We performed a retrospective cohort study of 1,891 consecutive patients at Samsung Medical Center in Seoul, South Korea, between March 2014 and January 2015^20^. Adult patients older than 20 years of age who had undergone spirometry and were referred to the pulmonology division for consultation prior to non-cardiothoracic surgery were included in this study. Patients with airflow obstruction (i.e., pre-bronchodilator FEV_1_/FVC < 0.7, n = 1,022) and those who received local anesthesia (n = 118), underwent heart and aorta surgery (n = 36), or had a prior lung resection (n = 34) were excluded, leaving 681 patients with a normal or restrictive spirometric pattern for inclusion in this study (Fig. 1). Patients were categorized into three groups, namely, those with (1) a normal spirometric pattern (n = 399), (2) a mild restrictive spirometric pattern (n = 169), and (3) a moderate-to-severe restrictive spirometric pattern (n = 113) based on the results of preoperative spirometry and who were followed over time to observe PPCs. The Institutional Review Board of the Samsung Medical Center (IRB no. 2016–09–080) approved this study and waived the consent of the study participants given the retrospective nature of the study. All methods were performed in accordance with the relevant guidelines and regulations.

### Definition and assessment of PPC

Patients were diagnosed with a PPC if they developed respiratory failure, pleural effusion, atelectasis, respiratory infection, pneumothorax, or bronchospasm within the first seven postoperative days. Each PPC was defined as follows^5,25,26^: respiratory failure: postoperative PaO_2_ < 60 mmHg, PaO_2_/FiO_2_ ratio < 300 or SpO_2_ < 90% that required oxygen therapy; pleural effusion: blunted costophrenic angle, loss of diaphragmatic silhouette, displacement of adjacent anatomical structures, or a hazy opacity in one hemithorax with preserved vascular shadow on chest radiograph, followed by confirmation with ultrasonography in some cases; atelectasis: lung opacification with a volume decrease; respiratory infection: antibiotic treatment for at least one of a new or changed sputum, new or changed lung opacities, fever, or leukocytosis; pneumothorax, as air in the pleural space; and bronchospasm, as newly detected expiratory wheezing treated with bronchodilators.

A routine chest X-ray was generally performed within 24 h after non-cardiothoracic surgery. Additional chest X-ray imaging was performed for suspected PPC occurrence at the discretion of the attending physician. Attending physicians assessed the PPC, and two authors (B. Shin and H. Lee), who were blinded to the spirometry results, confirmed whether the events fulfilled the definitions for each case.

### Preoperative PPC assessment and perioperative prevention strategy

Prior to surgery, which was performed under general anesthesia, all patients were evaluated using the Preoperative Pulmonary Complications Assessment tool^5,20^, a risk stratification scoring instrument based on age, the American Society of Anesthesiologists (ASA) physical status classification, smoking status, the presence or absence of airflow limitation, serum albumin level, chest radiographic findings, and surgical data (elective or emergency surgery, type of surgery, and duration of surgery). We selected items for the Preoperative Pulmonary Complications Assessment based on the American College of Physicians guidelines for risk assessment and strategies to reduce PPCs in patients undergoing non-cardiothoracic surgery^3,4^. During the perioperative period, we implemented a preoperative prevention strategy with incentive spirometry in every patient who underwent surgery under general anesthesia^27^. Patients were also encouraged to take deep inspirations, actively cough, and expectorate sputum during the postoperative period. We recommended that all smokers cease smoking for at least two weeks before surgery.

### Pulmonary function test

**Figure 2:**
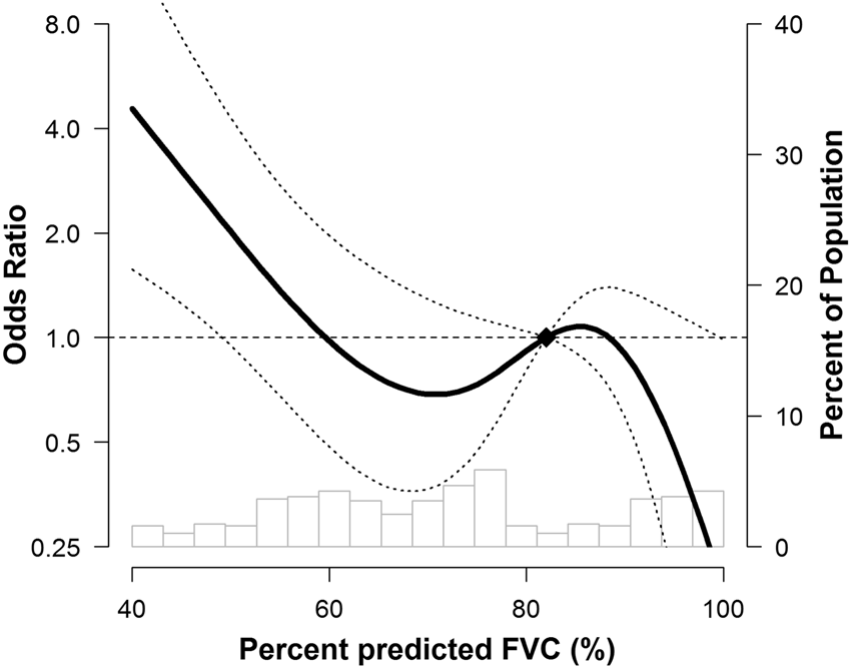
Multivariable-adjusted prevalence ratios (95% confidence interval) of percent predicted FVC. Percent predicted FVCs were modeled as restricted cubic splines with knots at the 5^th^, 35^th^, 65^th^, and 95^th^ percentiles of the sample distribution. Poisson regression models were adjusted for age, sex, body mass index, ASA physical status (<class 2 and ≥class 2), smoking status (current versus noncurrent smoker), presence of congestive heart failure (yes/no), presence of diabetes mellitus (yes/no), presence of chronic lung disease (yes/no), type of surgery (dichotomous categories for open abdominal surgery versus laparoscopic or other^†^ surgery), duration of surgery, and albumin (<4.0 g/dL and ≥4.0 g/dL). *ASA* American Society of Anesthesiologists, *FVC* forced vital capacity, *PPC* postoperative pulmonary complications. ^†^Includes breast, endocrine, vascular, orthopedic and spinal, gynecologic, urologic, ophthalmologic, and plastic surgery.

Preoperative spirometry was performed using a Vmax 22 apparatus (SensorMedics, Yorba Linda, CA, USA) according to American Thoracic Society/European Respiratory Society criteria^7^. All preoperative spirometry was performed in the pulmonary function lab. Spirometry was generally performed within one month before surgery. The percentages of predicted FEV_1_ and FVC were calculated using reference equations from a representative Korean sample^28^. A normal spirometric pattern was defined as no airflow obstruction (pre-bronchodilator FEV_1_/FVC ≥ 0.7) with FVC ≥ 80% predicted. A restrictive spirometric pattern was defined as no airflow obstruction (pre-bronchodilator FEV_1_/FVC ≥ 0.7) with a reduced FVC < 80% predicted^6^. The severity of a restrictive spirometric pattern was categorized as follows: mild was 60 ≤ FVC < 80% predicted and moderate-to-severe was FVC < 60% predicted. We determined an FVC of 60% predicted as a cut-off point since the incidence of PPCs increases abruptly from this point (Fig. 2).

### Statistical analysis

Data are presented as the mean (standard deviation [SD]), median (interquartile range [IQR]), or number (percentages) as appropriate. To test for linear trends, the group category was included as a continuous variable in the regression models. We used logistic regression to examine the associations between spirometric patterns and PPC risk. We adjusted for age, sex, body mass index (BMI), ASA physical status (<class 2 and ≥class 2), smoking status (current vs. noncurrent smoker), preoperative albumin (<4.0 g/dL and ≥4.0 g/dL), presence of congestive heart failure (yes/no), presence of diabetes mellitus (yes/no), presence of chronic lung disease (yes/no), type of surgery (dichotomous categories for open abdominal surgery vs. laparoscopic or other surgery), and duration of surgery. All data were analyzed using Stata (version 13.0; Stata Corporation, College Station, TX, USA).

### Ethics approval and consent to participate

The Institutional Review Board of the Samsung Medical Center (IRB no. 2016–09–080) approved this study and waived the consent of the study participants given the retrospective nature of the study. All methods were performed in accordance with the relevant guidelines and regulations.

## Results

### Patients

The study population included 339 men (49.8%) with a mean (SD) age of 66.4 (10.9) years. The mean (SD) BMI was 24.1 (3.8) kg/m^2^, and 30.3% were current or ex-smokers. Approximately 75% of study participants had an ASA physical status ≥class 2. The most common comorbidities included malignancy (46.7%), chronic lung disease (24.6%), diabetes mellitus (20.7%), and congestive heart failure (8.8%). The most common type of surgery was abdominal surgery (32.5%), including laparoscopic abdominal surgery (14.5%), followed by neurosurgery (23.1%) and head and neck surgery (7.5%). Emergent surgery was performed in 10 patients (1.5%). The median (IQR) duration of surgery was 2.3 (1.5–3.7) hours. The mean (SD) FEV_1_/FVC was 78.9 (5.8%). The mean FVC, L (% predicted) and FEV_1_, L (% predicted) were 2.84 L (80.1% predicted) and 2.24 L (86.1% predicted), respectively.

As shown in Table 1, older age, male sex, ASA physical status ≥2, ever smoking, lower albumin levels, diabetes mellitus, congestive heart failure, pulmonary infection, interstitial lung disease, advanced lung cancer, and neurosurgical procedures were all more common among those with more severe restrictive spirometric patterns. BMI and surgery duration were similar across the groups.

**Table 1:** Comparison of baseline characteristics of study participants across the severity of restrictive spirometric pattern^*^.

	Normal spirometric pattern (n = 399)	Mild restrictive spirometric pattern (n = 169)	Moderate-to-severe restrictive spirometric pattern (n = 113)
Age, years	64.8 (10.8)	69.0 (10.4)	68.1 (10.9)
Sex, male	177 (44.4)	93 (55.0)	69 (61.1)
Body mass index kg/m^2^	24.0 (3.4)	24.6 (3.9)	24.0 (4.8)
ASA physical status ≥class 2	285 (71.4)	130 (76.9)	98 (86.7)
Ever smoker	102 (25.6)	65 (38.5)	39 (34.5)
Preoperative albumin, g/dL	4.3 (0.4)	4.1 (0.5)	3.8 (0.7)
Comorbidities			
Diabetes mellitus	59 (14.8)	43 (25.4)	39 (34.5)
Congestive heart failure	19 (4.8)	20 (11.8)	21 (18.6)
Malignancy	187 (46.9)	80 (47.3)	51 (45.1)
Underlying lung disease	49 (12.3)	68 (40.2)	51 (45.1)
Current or previous pulmonary infection	34 (8.5)	35 (20.7)	27 (23.9)
Interstitial lung disease	9 (2.3)	17 (10.1)	5 (4.4)
Advanced lung cancer	6 (1.5)	16 (9.5)	19 (16.8)
Surgery			
Emergency surgery	6 (1.5)	3 (1.8)	1 (0.9)
Type of surgery			
Abdominal	119 (29.8)	49 (29.0)	53 (46.9)
Laparoscopic	50 (12.5)	28 (16.6)	21 (18.6)
Neurosurgery	107 (26.8)	40 (23.7)	10 (8.9)
Head and neck	30 (7.5)	13 (7.7)	8 (7.1)
Others^†^	143 (35.8)	67 (39.6)	42 (37.2)
Duration of surgery, h	2.8 (1.9)	2.7 (1.9)	2.6 (1.9)
Pulmonary function test			
FVC, L	3.24 (0.79)	2.55 (0.62)	1.88 (0.47)
FVC, % predicted	92.3 (10.1)	70.7 (5.5)	50.9 (7.3)
FEV_1_, L	2.55 (0.61)	1.98 (0.49)	1.51 (0.38)
FEV_1_, % predicted	97.9 (12.2)	77.3 (9.3)	57.2 (9.9)
SpO_2_, %	99.5 (1.4)	99.0 (2.0)	98.2 (2.7)

Data are presented as number (%) or mean with standard deviation.

*ASA* American Society of Anesthesiologists, *FVC* forced vital capacity, *FEV*_1_ forced expiratory volume in 1 s, *SpO*_2_ pulse oximetry

^*^The severity of restrictive spirometric pattern was categorized as follows; mild: 60 ≤ FVC < 80% predicted and moderate-to-severe: FVC < 60% predicted; ^†^includes breast, endocrine, vascular, orthopedic and spinal, gynecologic, urologic, ophthalmologic, and plastic surgery.

Missing values are included as follows: preoperative albumin (n = 1) and SpO_2_ (n = 7).

### PPC occurrence and type of surgery

**Figure 3:**
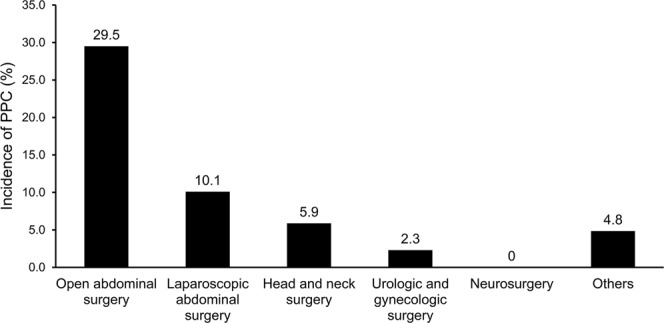
Incidence of postoperative pulmonary complications according to type of surgery. *PPC* postoperative pulmonary complications. Others include breast, endocrine, vascular, orthopedic and spinal, ophthalmologic, and plastic surgery.

During the study period, 59 (8.7%) study participants developed a PPC following non-cardiothoracic surgery. As shown in Fig. 3, the occurrence of PPCs was most frequent among those who underwent open abdominal surgery (29.5%, 36/122), followed by laparoscopic abdominal surgery (10.1%, 10/99), head and neck surgery (5.9%, 3/51), urologic and gynecologic surgery (2.3%, 2/87), and others including breast, endocrine, vascular, orthopedic and spinal, ophthalmologic, and plastic surgery (4.8%, 8/165).

### PPC occurrence in patients with normal or restrictive spirometric patterns

As shown in Table 2, the most common complication was respiratory failure (5.4%, 37/681), followed by atelectasis (5.1%, 35/681), pleural effusion (4.9%, 33/681), and respiratory infection (1.3%, 9/681). PPCs occurred more frequently in patients with a restrictive spirometric pattern (12.4%, 35/282) compared with patients with a normal spirometric pattern (6.0%, 24/399) (*P* = 0.003). Patients with a restrictive spirometric pattern were more likely to have respiratory failure (11.0% [31/282] vs. 1.5% [6/399], *P* < 0.001) and respiratory infection (2.5% [7/282] vs. 0.5% [2/399], *P* = 0.038) compared with those with a normal spirometric pattern. In contrast, atelectasis (6.7% [19/282] vs. 4.0% [16/399]) and pleural effusions (5.7% [16/282] vs. 4.3% [17/399]) occurred at similar rates between both groups.

**Table 2:** Associations between the severity of restrictive spirometric pattern^*^ and the risk of postoperative complications.

	Normal spirometric Pattern (n = 399)	Restrictive spirometric pattern			*P v*alue for trend
		Mild (n = 169)	Moderate-to-severe (n = 113)	Total (n = 282)	
No (%) of events	24 (6.0)	11 (6.5)	24 (21.2)	35 (12.4)	
Respiratory failure	6 (1.5)	9 (5.3)	22 (19.5)	31 (11.0)	<0.001
Atelectasis	16 (4.0)	5 (3.0)	14 (12.4)	19 (6.7)	0.005
Pleural effusion	17 (4.3)	5 (3.0)	11 (9.7)	16 (5.7)	0.068
Respiratory infection	2 (0.5)	3 (1.8)	4 (3.5)	7 (2.5)	0.019
Unadjusted odds ratio (95% CI)	*Reference*	1.09 (0.52–2.27)	4.21 (2.29–7.76)	2.21 (1.29–3.81)	<0.001
Adjusted odds ratio (95% CI)	*Reference*	0.95 (0.40–2.24)	2.64 (1.22–5.67)	1.28 (0.92–1.80)	0.017
	**Odds ratio per 5% decrease in FVC (95% CI)**				***P*** **value**
Unadjusted odds ratio (95% CI)	1.23 (1.14–1.32)				<0.001
Adjusted odds ratio (95% CI)	1.18 (1.08–1.30)				<0.001

Adjusted for age, sex, body mass index, ASA physical status (<class 2 and ≥class 2), smoking status (current versus noncurrent smoker), presence of congestive heart failure (yes/no), presence of diabetes mellitus (yes/no), presence of chronic lung disease (yes/no), type of surgery (open abdominal surgery versus laparoscopic or other surgery), duration of surgery, and albumin (<4.0 g/dL and ≥4.0 g/dL).

*FVC* forced expiratory volume, *CI* confidence interval, *ASA* American Society of Anesthesiologists.

^*^Severity of restrictive spirometric pattern was categorized as follows; mild: 60 ≤ FVC < 80% predicted and moderate-to-severe: FVC < 60% predicted.

### Severity of restrictive spirometric pattern and PPC risk

The occurrence of PPCs was markedly higher in patients with a moderate-to-severe restrictive spirometric pattern (21.2%) compared with those with mild restrictive (6.5%) and normal spirometric patterns (6.0%) (*P* for trend test < 0.001). We also evaluated the occurrence of each type of PPC across the severity of restrictive spirometric patterns. The incidences of respiratory failure, atelectasis, pleural effusion, and respiratory infection increased in patients with a moderate-to-severe restrictive spirometric pattern compared with those with normal spirometric patterns (19.5% vs. 1.5%, 12.4% vs. 4.0%, 9.7% vs. 4.3%, and 3.5% vs. 0.5%, respectively).

The crude and adjusted odds ratio (OR) for the occurrence of PPCs in patients with mild or moderate-to-severe restrictive spirometric patterns undergoing non-cardiothoracic surgery are shown in Table 2. While patients with a mild restrictive spirometric pattern did not have an increased risk of PPCs, regardless of the adjustments made for confounding factors (unadjusted OR = 1.09, 95% confidence interval [CI] = 0.52–2.27; adjusted OR = 0.95, 95% CI = 0.40–2.24), those with a moderate-to-severe restrictive spirometric pattern had a significantly increased risk of PPCs in both the crude model and the adjusted model (unadjusted OR = 4.21, 95% CI = 2.29–7.76; adjusted OR = 2.64, 95% CI = 1.22–5.67). When FVC (% predicted) was analyzed as a continuous variable, the prevalence ratio of PPCs from a fully adjusted model increased at the point around an FVC of 60% predicted (Fig. 2), i.e., the point where the moderately restrictive spirometric pattern was defined.

In addition, the secondary analyses of groups with open or laparoscopic abdominal surgery, those without abdominal surgery and groups with BMI > 25 (kg/m^2^) or ≤25 (kg/m^2^), showed no interaction in the fully adjusted models (Table 3). Compared with patients with a normal spirometry, those with a moderate-to-severe restrictive spirometric pattern were 14.49 (95% CI 1.92–197.75), 6.14 (95% CI, 1.72–21.93), and 4.33 (95% CI 1.15–16.22) times more likely to develop a PPC when they underwent laparoscopic abdominal surgery or non-abdominal surgery or were obese, respectively. However, there was no significant difference in the RR for the occurrence of PPCs across the severity of restrictive spirometric patterns among the patients who underwent open abdominal surgery or those who were non-obese.

**Table 3:** Association between the severity of restrictive spirometric pattern^*^ and the risk of postoperative complications according to abdominal surgery and obesity.

	Adjusted OR (95% CI)	*P* for interaction
**Abdominal surgery**		0.039
**Non-abdominal surgery (n = 460)**		
Normal	*Reference*	
Mild restrictive spirometric pattern	0.40 (0.05–3.45)	
Moderate-to-severe restrictive spirometric pattern	6.14 (1.72–21.93)	
**Open abdominal surgery (n = 122)**		
Normal	*Reference*	
Mild restrictive spirometric pattern	0.70 (0.19–2.54)	
Moderate-to-severe restrictive spirometric pattern	1.03 (0.36–2.94)	
**Laparoscopic abdominal surgery (n = 99)**		
Normal	*Reference*	
Mild restrictive spirometric pattern	9.59 (0.91–100.97)	
Moderate-to-severe restrictive spirometric pattern	19.49 (1.92–197.75)	
**Obesity**		
**Non-obese (n = 421)**		0.67
Normal	*Reference*	
Mild restrictive spirometric pattern	0.85 (0.30–2.37)	
Moderate-to-severe restrictive spirometric pattern	2.24 (0.27–5.60)	
**Obese (n = 260)**		
Normal	*Reference*	
Mild restrictive spirometric pattern	1.24 (0.57–2.67)	
Moderate-to-severe restrictive spirometric pattern	4.33 (1.15–16.22)	

Adjusted for age, sex, body mass index, American Society of Anesthesiologists physical status (<class 2 and ≥class 2), smoking status (current versus noncurrent smoker), presence of congestive heart failure (yes/no), presence of diabetes mellitus (yes/no), presence of chronic lung disease (yes/no), type of surgery (dichotomous categories for open abdominal surgery versus laparoscopic or other surgery), duration of surgery, and albumin (<4.0 g/dL and ≥4.0 g/dL).

^*^Severity of restrictive spirometric pattern was categorized as follows; mild: 60 ≤ FVC < 80% predicted and moderate-to-severe: FVC < 60% predicted;

^†^Obesity was defined as body mass index > 25 kg/m^2^.

### Severity of restrictive spirometric pattern and length of hospital stay and overall mortality

The length of hospital stay increased across restrictive spirometric pattern categories: median (IQR) 9.0 (6.0–11.0) days for patients with normal spirometry vs. 9.0 (6.0–15.0) days for patients with a mild-restrictive spirometric pattern vs. 11.0 (8.0–22.0) days for patients with moderate-to-severe spirometry; *P* for trend = 0.002. The 30-day and 90-day mortality rates increased across the categories as well: 0.3% and 2.0% for patients with normal spirometry vs. 2.4% and 7.7% for patients with a mild restrictive spirometric pattern vs. 3.5% and 8.8% for patients with a moderate-to-severe restrictive spirometric pattern, respectively; *P* for trend = 0.008 for 30-day mortality and 0.001 for 90-day mortality. However, among the 59 patients who developed PPCs, there were no trends in the length of stay or 30- and 90-day mortality rates across the severity of restrictive spirometric patterns (Table 4).

**Table 4:** Length of hospital stay, and 30-day and 90-day mortality of study participants.

	Normal spirometric pattern	Mild restrictive spirometric pattern	Moderate-to-severe restrictive spirometric pattern	*P* value for trend
Overall (n = 681)				
Length of stay, days	9.0 (6.0–11.0)	9.0 (6.0–15.0)	11.0 (8.0–22.0)	0.002
30-day mortality	1 (0.3)	4 (2.4)	4 (3.5)	0.008
90-day mortality	8 (2.0)	13 (7.7)	10 (8.8)	0.001
Among those with a PPC (n = 59)				
Length of stay, days	11.0 (9.5–16.5)	25.0 (10.0–49.0)	15.0 (10.0–21.5)	0.916
30-day mortality	0	0	2 (8.3)	0.980
90-day mortality	3 (12.5)	3 (27.3)	5 (20.8)	0.461

Data are presented as number (%) or median (interquartile range). ^*^Severity of restrictive spirometric pattern was categorized as follows; mild: 60 ≤ FVC < 80% predicted and moderate-to-severe: FVC < 60% predicted.

*PPC* postoperative pulmonary complications.

## Discussion

In this study, we found that the incidence of PPCs in patients with a restrictive spirometric pattern was higher than that of patients with normal spirometry, and this was most evident in patients with a moderate-to-severe restrictive spirometric pattern. We also showed that a moderate-to-severe restrictive spirometric pattern was independently associated with the occurrence of PPCs following non-cardiothoracic surgery. To the best of our knowledge, this is the first study to comprehensively show an independent association between a restrictive spirometric pattern and PPCs in patients who underwent various types of non-cardiothoracic surgery.

Overall, we found a 12.4% incidence of PPCs in patients with a restrictive spirometric pattern. Considering that the incidence of PPCs in patients with airflow limitations or COPD ranges from 11–24%^2,5,19,20,25,29^, our study suggests that the PPC burden among patients with restrictive spirometric pattern has been largely unrecognized, and more attention should be given to this patient group. Historically, a restrictive spirometric pattern has been considered to be linked to interstitial lung disease, neurologic disorders, and space-occupying lesions such as tumors or effusions^7^. However, there is accumulating evidence that many common medical ailments, such as aging, smoking, obesity, diabetes mellitus, and cardiovascular disease, are also associated with a restrictive spirometric pattern^9,15,16,18,30,31^, which is also shown in this study. Although there is a possibility that these comorbidities affected the occurrence of PPCs in patients with a restrictive spirometric pattern, the PPC risk persisted after adjusting for these comorbidities. Thus, we suggest that a restrictive spirometric pattern itself is associated with an increased risk of PPCs.

Despite the recent interest in patients with restrictive spirometric patterns, only a few studies have evaluated the relationship between a restrictive spirometric patterns and PPCs. A previous study revealed that in addition to a low FEV_1_ (<1 L/min), a low FVC (<1.5 L/min) is also significantly associated with PPCs after non-thoracic surgery^2^. Another study evaluated pre-operative variables associated with PPCs in patients who underwent abdominal surgery, of whom 62% patients had restrictive spirometric pattern. In that study, patients who developed PPCs had lower FVC and FEV_1_ compared with those who did not develop PPCs^32^. However, since a low FVC can also occur in patients with airflow limitations, the patients in these studies cannot be definitively classified as having a restrictive spirometric pattern, although it is highly likely that they did. Furthermore, the findings from these studies are limited, because of the relatively small sample sizes^2,32^. Recently, Kim and colleagues^19^ evaluated PPCs in patients who underwent abdominal surgery and showed that the prevalence of PPCs in patients with a restrictive spirometric pattern was 18%. In that study, a restrictive spirometric pattern was not independently associated with PPCs. However, the evaluation of the association between a restrictive spirometric pattern and PPCs was not the main purpose of that study. There were also other limitations in that study, namely, the relatively small number of patients with a restrictive spirometric pattern, the confinement of PPC evaluations to patients who underwent abdominal surgery, and the lack of consideration of the effect of the severity of the restrictive spirometric pattern on the occurrence of PPCs. Other studies have shown that a reduced FVC is significantly associated with the occurrence of PPCs in both pediatric and adult patients with scoliosis^21–23,33,34^. However, those studies have limitations that the study population was confined to patients with scoliosis, and the number of study population was relatively small. Our study overcomes these limitations by having a relatively larger number of patients with a restrictive spirometric pattern who underwent a variety of surgeries. We also evaluated the effect of the severity of the restrictive spirometric pattern on the occurrence of PPCs. Thus, we provide more generalizable evidence that restrictive spirometric pattern is associated with an increased risk of PPCs in patients undergoing non-cardiothoracic surgery.

Another important finding of our study is that the presence and severity of the restrictive spirometric pattern is associated with higher 30- or 90-day mortalities and a longer length of hospital stay in patients undergoing non-cardiothoracic surgery, consistent with the findings of previous studies^35,36^. The 30- and 90-day mortality rates significantly increased as the severity of restrictive spirometric pattern progressed, suggesting that a restrictive spirometric pattern can be an important biomarker associated with postoperative mortality in patients undergoing non-cardiothoracic surgery.

Despite evidence of a causal link between a restrictive spirometric pattern, PPCs and mortality following non-cardiothoracic surgery, the exact underlying mechanisms are largely unknown. The impact of restrictive spirometric pattern on PPCs may in part be due to the restrictive physiology imposed on the respiratory system; the potential reduction in lung compliance during the postoperative period due to postoperative respiratory depression and/or postoperative pain can lead to atelectasis, respiratory failure, and respiratory infection. Interestingly, in our study, obese patients or those who underwent non-abdominal or laparoscopic abdominal surgery were especially prone to PPCs. The authors cautiously suggest that restrictive physiology during the postoperative period may be more profound in obese patients. In addition, the impact of open abdominal surgery alone on the development of PPCs may be more pronounced than that of the restrictive spirometric pattern itself. Considering the limitations of the subgroup analyses regarding this issue, more investigations are needed in future studies.

Chronic debilitating conditions associated with a restrictive spirometric pattern may also have influenced the increased postoperative mortality in this study. Regarding this association, it is worth noting that subjects with a restrictive spirometric pattern generally had a poor medical prognosis, with a higher burden of comorbidities. A long-term, large, population-based cohort study also found that patients with a recurrent restrictive pattern were at higher risk of mortality due to heart disease, stroke, and diabetes mellitus^15^. Additionally, patients with a restrictive spirometric pattern were more likely to have dyspnea, poor health, decreased exercise capacity, and increased all-cause mortality^8,15^, conditions that are comparable to the problems experienced by patients with moderate COPD^9^.

This study has several limitations. First, this study was conducted in a single referral hospital. Thus, the patients with a restrictive spirometric pattern in this study had a higher burden of advanced comorbidities, such as lung cancer and congestive heart failure, than would be expected in the general population. Although we adjusted for these comorbidities, the higher burden of these advanced comorbidities may have affected the restrictive spirometric pattern. Thus, the generalizability of our findings may be limited and may not apply to patients with a restrictive spirometric pattern who are otherwise healthy. Second, due to the observational nature of this study, we could not control some potential confounders, such as operative and postoperative care, between the patients with restrictive and normal spirometric patterns. However, a routine perioperative preventive strategy was universally applied in our hospital for all patients who underwent spirometry, irrespective of the results. Interventions such as a consultation with respiratory care practitioners were selectively introduced with the development of early signs of a PPC. Thus, although we were not able to quantify the postoperative management, we assume that adjusting those variables would possibly highlight the differences in outcome. Third, we did not routinely measure lung volume or diffusing capacity, which may have provided information about the etiologies of the restrictive spirometric pattern^7^. Lung volume and diffusing capacity measurements require additional costs and time and are not routinely performed in regular clinical practice. Because the purpose of performing pulmonary function tests is to screen for patients who have a high risk of developing PPCs, we cautiously suggest that spirometry might be adequate for this purpose. Fourth, although we strongly recommended perioperative management, such as smoking cessation before surgery, as well as deep inspiration, active coughing, and sputum expectoration during the perioperative period, we were not able to assess whether patients actually followed these recommendations. Lastly, the number of patients with interstitial lung disease was relatively small. Thus, we could not elucidate whether patients with a restrictive spirometric pattern associated with interstitial lung diseases have a higher risk of PPCs compared with those with a restrictive spiroemtric pattern associated with other etiologies.

## Conclusions

We found a significant association between a restrictive spirometric pattern and PPCs in patients undergoing non-cardiothoracic surgery. Our study suggests that a moderate-to-severe restrictive spirometric pattern should be regarded as a risk factor for PPC.

## Data Availability

All data extracted in this study are included in this article.
